# Unprocessed Meat Consumption and Incident Cardiovascular Diseases in Korean Adults: The Korean Genome and Epidemiology Study (KoGES)

**DOI:** 10.3390/nu9050498

**Published:** 2017-05-15

**Authors:** Kyong Park, Jakyung Son, Jiyoung Jang, Ryungwoo Kang, Hye-Kyung Chung, Kyong Won Lee, Seung-Min Lee, Hyunjung Lim, Min-Jeong Shin

**Affiliations:** 1 Department of Food and Nutrition, Yeungnam University, Gyeongsan, Gyeongbuk 38541, Korea; kypark@ynu.ac.kr (K.P.); jakyungson@ynu.ac.kr (J.S.); today@ynu.ac.kr (J.J.); 2 Korea National Enterprise for Clinical Trials, Seoul 04143, Korea; wooo@yonsei.ac.kr; 3 Severance Institute for Vascular and Metabolic Research, Yonsei University College of Medicine, Seoul 06273, Korea; chk@yuhs.ac; 4 Department of Public Health Sciences, BK21PLUS Program in Embodiment: Health-Society Interaction, Graduate School, Korea University, Seoul 02841, Korea; chirstkw@naver.com; 5 Department of Food and Nutrition, Sungshin Women’s University, Seoul 01133, Korea; smlee@sungshin.ac.kr; 6 Department of Medical Nutrition, Graduate School of East-West Medical Science, Kyung Hee University, Yongin 17104, Korea; hjlim@khu.ac.kr

**Keywords:** unprocessed meat, cardiovascular disease, cohort, Asian

## Abstract

Meat consumption has been shown to be associated with cardiovascular disease (CVD) risk in Western societies; however, epidemiological data are limited on the Korean population. Therefore, we examined the associations between unprocessed meat consumption and CVD incidence in Korea. Data were derived from the Ansung-Ansan cohort (2001–2012), including 9370 adults (40–69 years) without CVD or cancer at baseline. Total unprocessed meat consumption was estimated as the sum of unprocessed red meat (beef, pork, and organ meat) and poultry consumption. In the fully adjusted Cox regression model, the relative risks of CVD across increasing quintiles of total unprocessed meat intake were 1.0 (reference), 0.72 (95% confidence interval (CI): 0.55, 0.95), 0.57 (95% CI: 0.42, 0.78), 0.69 (95% CI: 0.51, 0.95), and 0.69 (95% CI: 0.48, 0.97), but no significant linear trend was detected (*p* for trend = 0.14). Frequent poultry consumption was significantly associated with a decreased CVD risk; this association showed a dose-response relationship (*p* for trend = 0.04). This study showed that a moderate intake of total unprocessed meat was inversely associated with CVD risk. A significant inverse association between poultry consumption and incident CVD was observed in Korean adults, requiring further confirmation in other populations.

## 1. Introduction

The prevalence of cardiovascular disease (CVD) is high in both developed and developing countries; hence, it is crucial to identify dietary risk factors [1]. Considering the substantial burden of CVD in Korea [2], effective strategies are urgently needed to achieve the public health goal of disease prevention. 

Numerous studies have attempted to establish an association between meat intake and CVD incidence and mortality [3,4,5,6,7,8,9,10]. A recent meta-analysis of studies that were mainly conducted in Western countries demonstrated a relationship between high meat consumption and increased cardiovascular mortality [5,6,7]. Processed meat intake was also found to be associated with a higher incidence of coronary heart disease (CHD) in 17 prospective studies and three case-control studies including 1,218,380 individuals from the US, Europe, Australia, and Asia [10]. The results of a systematic review and meta-analysis including data from prospective cohort studies and randomized controlled trials have shown that the intake of processed meats was associated with a 42% higher risk of CHD and 19% higher risk of diabetes in adults [10]. On the other hand, white meat has been considered a healthy replacement for unprocessed red meat in terms of CVD risk. Low consumption of poultry was associated with increased all-cause and cardiovascular mortality when compared to moderate consumption in cohorts in the US and Europe [3]. As evidenced by the Nurses’ Health Study [11], higher consumption of poultry meat was related to a lower risk of coronary heart disease in women when compared to higher consumption of red meat. Similarly, a Chinese cohort study demonstrated suggestive inverse associations between poultry intake and the risk for total and cardiovascular mortality in men [12]; other studies reported similar results [13,14]. In line with these results, the new US Dietary Guidelines (8th edition, 2015–2020) recommend “healthy eating patterns” that include the intake of lean meats and poultry (26 ounce-equivalents of meat, poultry, and eggs per week at the 2000-calorie level) to stay healthy [15]. Most previous studies reported that meat consumption is a risk factor for CVD mainly because of its high saturated fat and cholesterol content [16]. However, there has been limited information on the association between meat consumption and cardiovascular health in the Korean population. In the present study, we therefore aimed to evaluate whether the consumption of total and specific types of unprocessed meats was associated with incident CVD in the Ansung-Ansan cohort (2001–2012) of Korean adults.

## 2. Materials and Methods

### 2.1. Study Population

The Ansung-Ansan cohort is an ongoing prospective cohort study in South Korea. Detailed information on its study design and protocol can be found elsewhere [17,18]. Briefly, a baseline survey was conducted in 2001–2002, and 10,030 adults aged 40–69 years were enrolled from the two communities of Ansan (urban region) and Ansung (rural region) in South Korea. Five follow-up examinations were conducted in 2003–2004, 2005–2006, 2007–2008, 2009–2010, and 2011–2012.

At each examination, data on demographic and lifestyle characteristics, metabolic and cardiovascular profiles, medical history, and disease incidence were collected. Procedures were conducted by trained interviewers who followed standardized protocols. Participants with a history of CVD or those taking CVD-related medications (*n* = 276), those with a history of cancer (*n* = 222), and those with an implausible energy intake (<500 kcal/day or >5000 kcal/day, *n* = 162) [19] were excluded based on baseline information. Thus, 9370 participants were included in the final analysis.

Informed consent was obtained from all individual participants included in the study, and the study was approved by the ethics committee of the Korean Centers for Disease Control & Prevention’s and the Korea University Institutional (KU-IRB-15-EX-256-A-1) review boards.

### 2.2. Dietary Assessment

Dietary information was examined at baseline and at the second follow-up examination in 2005–2006. Usual dietary intake was assessed using a 110-item semi-quantitative food frequency questionnaire (SQFFQ). The validity and reproducibility of the SQFFQ has been described elsewhere [20]. The SQFFQ includes 9 categories for food frequency, from never/seldom to ≥3 times/day and 3 options for portion size (0.5 times the reference, reference, and 1.5–2 times the reference) for each item. For instance, portion size options for pork belly were 75 g (0.5 times the reference), 150 g (reference), and 225 g (1.5 times the reference).

For our analysis, a standardized intake frequency that considered both frequency and portion size was calculated. To minimize misclassification of dietary information, we used average intake values of food items reported at baseline and the second follow-up examination (2005–2006). For missing dietary variables at the second follow-up, data was imputed using the fully conditional specification approach [21].

Unprocessed red meat consumption was defined as the sum of the weekly consumption of beef (roast beef, beef ribs, and beef soup), pork (pork belly, steamed pork, and roast pork), and organ meat. Total unprocessed meat consumption was estimated as the consumption of unprocessed red meat plus unprocessed poultry (chicken legs, chicken wings, and other chicken meat). Total vegetable consumption was calculated as the sum of the weekly consumption of Chinese cabbage, spinach, lettuce, perilla leaves, balloon flower roots, bean sprouts, bracken, pepper leaves, crown daisies, cucumbers, carrots, onions, green peppers, aged amber, and oyster mushrooms. Total fruit consumption was estimated as the sum of the weekly consumption of persimmons, mandarins, oriental melons, bananas, pears, apples, oranges, watermelons, peaches, strawberries, and grapes.

### 2.3. General Characteristics and Anthropometric Measurements

Survey questionnaires were administered by trained interviewers to obtain demographic (age, sex, residential area, educational level, and household income) and behavioral (smoking, alcohol consumption, and physical activity) information at each examination visit. Educational levels were categorized as elementary school graduation or below, middle school graduation, high school graduation, and college graduation or above. Monthly household income was categorized into 4 groups, from <1,000,000 Korean won to ≥4,000,000 Korean won. Smoking status was categorized as never, former or current, and pack-years were calculated for current smokers. Alcohol consumption was classified as current alcohol consumption versus non-drinking. Among drinkers, weekly alcohol consumption was calculated using the frequency and amount of alcohol that was consumed. Physical activity levels were calculated as metabolic equivalents (METs)-h/week [22]. Height was measured within 0.1 cm (without shoes), and weight was measured in kg to the nearest of 0.01 kg (in light clothing and without shoes) by trained technicians. Body mass index (BMI) was calculated as weight in kilograms divided by height squared in meters. BMI cutoff values from the World Health Organization for Asian populations were applied [23].

### 2.4. Cardiovascular Disease Evaluation

Incident CVD cases were identified by biennial questionnaires, and all reported cases were confirmed through repeated in-depth personal interviews. CVD mortality information was not obtained for this analysis. CVD events were defined as newly diagnosed cases by a physician or being prescribed CVD-related medications. Cases included myocardial infarction, coronary artery disease, congestive heart failure, and/or stroke.

### 2.5. Statistical Analysis

Study participants were grouped by quintiles of meat intake (for total unprocessed meat, unprocessed red meat, and types of meat) to examine if there was a monotonically increasing or decreasing pattern of risk for CVD. Baseline characteristics were compared among quintiles of total unprocessed meat intake using Chi-square statistics for categorical variables and generalized linear regression with *p* for trend for continuous variables. Potential confounding variables were considered based on a preliminary analysis and a review of the related literature. Possible effect modification was determined for multiple demographic and lifestyle variables using multiplicative terms in the regression model; no significant effect modifier was observed. To systematically account for potential confounding factors, three covariate models using Cox proportional hazard regression were fitted to the data. Model 1 was adjusted for age, model 2 was additionally adjusted for sex, educational level, household income, residential area, smoking status, alcohol intake, BMI, physical activity, and total energy intake levels, and model 3 was further adjusted for total fruit and vegetable intake. To test for a linear trend across quintiles of meat consumption, a continuous variable using the median value of each quintile of meat intake was created. SAS version 9.3 (SAS institute, Cary, NC, USA) was used for all analyses, and a two-sided *p* value < 0.05 was considered statistically significant.

## 3. Results

The baseline characteristics of the study participants are presented according to quintiles of total unprocessed meat intake (Table 1). Participants with higher intakes of total unprocessed meats showed higher numbers of current smokers and alcohol drinkers, had higher incomes and educational levels, and consumed more fruits and vegetables when compared to those with lower intakes of unprocessed meats.

During the median follow-up of 7.8 years, 486 incident cases of CVD were identified. Intakes of total unprocessed meat and total unprocessed red meat were inversely associated with CVD incidence (Table 2). In the age-adjusted model (model 1), a significantly decreased risk of CVD was observed with each increasing quintile of total unprocessed meat intake (*p* for trend = 0.049). In the fully adjusted Cox regression model (model 3), the RRs for CVD across increasing quintiles of total unprocessed meat intake were 1.0 (reference), 0.72 (95% confidence interval (CI) 0.55–0.95), 0.57 (95% CI: 0.42–0.78), 0.69 (95% CI: 0.51–0.95), and 0.69 (95% CI: 0.48–0.97), but no significant linear trend was detected (*p* for trend = 0.14). Similarly, an inverse relationship was observed between unprocessed red meat intake and incident CVD, but the comparison between the lowest and highest quintiles of unprocessed red meat intake was not statistically significant (relative risk (RR) 0.79, 95% CI: 0.57–1.11), and no significant linear trend was detected (*p* for trend = 0.38) in the fully adjusted model (model 3).

Regarding specific types of unprocessed meat, the intake of beef and organ meat was not associated with an increased incidence of CVD. However, significant associations were observed between pork and poultry intake and the incidence of CVD for specific intake quintiles (Table 3). Moderate pork intake (1.90 servings per week, median value) was protective against CVD; this association was evident for the 4th quintile (RR: 0.60, 95% CI: 0.43, 0.83; model 3) when compared to the lowest quintile. However, the test for linear trend was not significant (*p* for trend = 0.38). Frequent poultry consumption was significantly associated with a decreased risk for CVD, with the association showing a dose-response relationship for all statistical models (*p* for trend = 0.04). In the fully adjusted model (model 3), participants having the highest quintile of poultry intake were 1.5 times less likely to develop CVD than those in the lowest quintile (95% CI: 0.47, 0.99).

## 4. Discussion

Our data revealed that Korean adults with a moderate consumption of total unprocessed meat (as measured by the sum of unprocessed red meat and poultry) and unprocessed red meat (as measured by the sum of beef, pork, and organ meat) had a lower incidence of CVD than those with the lowest level of consumption. Regarding unprocessed red meat consumption, the significance disappeared in the 5th quintile after adjustments for covariates, indicating that unprocessed red meat intake of more than five servings per week likely does not have a beneficial association with cardiovascular health in the Korean population.

When we performed a stratified analysis by type of meat, individuals in the higher quintile of poultry intake showed lower incident CVD than those in the lowest quintile. Many studies have proposed that the biological pathway by which unprocessed red meat increases CVD risk is based on red meat being a source of saturated fat and cholesterol, which are key factors in the atherosclerotic process and for cardiovascular risk (e.g., hypertension, abnormal lipid profile, and metabolic syndrome) [24]. Moreover, unprocessed red meat is a significant source of heme iron, which has been positively associated with oxidative stress and inflammation [25]. In line with this, most studies conducted in North America [3,5] and Europe [4] have reported positive associations between unprocessed red meat consumption and cardiovascular mortality (hazard ratio (HR): 1.18, 95% CI: 1.13, 1.23 in North America; HR: 1.14, 95% CI: 1.01, 1.28 in Europe).

However, results obtained from Asian studies have been conflicting [26,27]. A community-based prospective cohort study found an inverse association between unprocessed red meat c consumption and ischemic heart disease mortality in Japanese adults (HR: 0.66, 95% CI: 0.45, 0.97) [27]. A pooled analysis of eight Asian prospective studies reported that high red meat consumption showed an association with a reduced risk for cardiovascular mortality in both men and women [26]. Consistent with these findings, moderate intake of unprocessed red meat was inversely associated with incident CVD in our study. 

Inconsistent results between prior studies conducted in the US and Europe and the current study might be partly due to differences in the actual amounts of meat that was consumed by the participants of this cohort. Indeed, there are distinct differences in the amount of meat consumed among populations in East Asia, Europe, America, and the Near East/North Africa [28]. According to a report of the Organization for Economic Cooperation and Development and the United Nations Food and Agriculture Organization, beef and veal consumption was estimated to be 24.7 kg per capita and 14.0 kg per capita in in the US and OECD countries, respectively [29]. In contrast, beef and veal consumption was estimated to be 9.6 kg per capita in South Korea in 2015; this is significantly lower than that of residents of Western countries. In addition, differences in cooking methods might explain the inconsistent association between meat intake and the risk for CVD; food preparation, consumption and preference differ by culture [30]. However, we cannot exclude the possibility that certain biological pathways that differ by ethnicity might explain the differences in the associations between meat consumption and incident CVD among these populations; this requires further investigation.

Poultry is considered one of the healthier alternatives to unprocessed red meat and has been inversely associated with a risk for cardiovascular mortality [4,13] and incident CVD [12,15]. Consistent with earlier findings from a pooled analysis of an Asian population [26], we observed a significant inverse association between poultry intake and incident CVD in Korean adults. One of the biological mechanisms underlying the beneficial effect of poultry on cardiovascular health is that carnosine (which is abundant in chicken meat) functions as a free radical scavenger and displays anti-oxidative activity [31]. Carnosine was also reported to accelerate the metabolism of stress hormones such as cortisol and noradrenaline under stress, resulting in a decrease in the severity of oxidative stress [32]. Consequently, carnosine can reduce cellular damage and the harmfulness of oxidative stress, thereby exerting a protective effect against CVD. 

The current study has several strengths. First, we analyzed data from a long-term follow-up cohort, which enhances the reliability of the results. Second, we used repeated measurements of diet (at baseline and at the 2nd follow-up visit) to reduce measurement error. Last, various types of meats were examined in this study; thus, we were able to evaluate which type of meat contributed most significantly to the relationship between meat consumption and CVD risk.

Our study also has some limitations. Although we adjusted for multiple confounding factors including socioeconomic status, behavioral determinants, and other dietary factors, there might be unmeasured or unknown residual confounding factors that might have affected our results. Although we used the standardized intake frequencies, accounting for both frequency and portion size, servings per week calculated by standardized intake frequencies may not be directly comparable to grams of food items consumed since the portion sizes of each food item are different. Further, we were not able to obtain specific information on the cooking methods used by the study participants. Finally, processed meat consumption was not included in our results as the average consumption was too low.

## 5. Conclusions

In conclusion, we found that moderate consumption of total unprocessed meat showed an inverse association with incident CVD in a large, prospective, population-based cohort study of middle-aged Koreans. In particular, a significant inverse dose-dependent association between poultry consumption and incident CVD was observed. Further studies should examine the associations between substituting red meat for other sources of animal protein, such as poultry and fish, and incident CVD. Our findings require further confirmation in other populations whose meat intake is lower than the global average. 

## Figures and Tables

**Table 1:** Demographic and lifestyle characteristics of study participants according to quintiles of total unprocessed meat consumption in Korean adults ^1^.

	Frequency of Consumption (Quintile)				
	Q1	Q2	Q3	Q4	Q5
	(*n* = 1862)	(*n* = 1865)	(*n* = 1863)	(*n* = 1859)	(*n* = 1862)
Median of total unprocessed meat consumption, servings/week	0.5	1.1	1.9	3.1	5.5
Age, years	56.9 ± 0.2	53.2 ± 0.2	51.2 ± 0.2	49.8 ± 0.2	49.3 ± 0.2
Sex, *n* (%)					
Men	474 (25.5)	766 (41.1)	918 (49.3)	1077 (57.9)	1226 (65.8)
Women	1388 (74.5)	1099 (58.9)	945 (50.7)	782 (42.1)	636 (34.2)
Body mass index (BMI), kg/m^2^	24.6 ± 0.1	24.5 ± 0.1	24.6 ± 0.1	24.5 ± 0.1	24.8 ± 0.1
Current smokers, *n* (%)	289 (15.7)	395 (21.4)	467 (25.4)	553 (30.0)	693 (37.6)
Alcohol consumption (yes), *n* (%)	504 (27.3)	761 (41.1)	901 (48.6)	1099 (59.5)	1153 (62.3)
Education level, *n* (%)					
Elementary school graduation or lower	1073 (58.2)	709 (38.2)	539 (29.0)	398 (21.5)	350 (18.9)
Middle school graduation	359 (19.5)	443 (23.9)	473 (25.5)	422 (22.8)	435 (23.5)
High school graduation	313 (17.0)	492 (26.5)	590 (31.8)	710 (38.3)	714 (38.6)
College graduation or higher	100 (5.4)	211 (11.4)	254 (13.7)	322 (17.4)	351 (19.0)
Residential area, *n* (%)					
Ansan (urban)	633 (34.0)	872 (46.8)	1004 (53.9)	1187 (63.9)	1013 (54.4)
Ansung (rural)	1229 (66.0)	993 (53.2)	859 (46.1)	672 (36.2)	849 (45.6)
Monthly household income (KRW), *n* (%)					
<1,000,000	1073 (58.9)	745 (40.6)	572 (31.2)	402 (21.8)	421 (22.9)
1–<2,000,000	415 (22.8)	558 (30.4)	566 (30.8)	608 (33.0)	552 (30.0)
2–<4,000,000	277 (15.2)	429 (23.4)	555 (30.2)	648 (35.2)	657 (35.7)
≥4,000,000	57 (3.1)	103 (5.6)	143 (7.8)	183 (9.9)	210 (11.4)
Physical activity, *n* (%) ^3^					
Low	992 (55.1)	1093 (60.1)	1146 (63.5)	1215 (67.3)	1102 (61.6)
Mid	290 (16.1)	304 (16.7)	305 (16.9)	303 (16.8)	328 (18.3)
High	517 (28.7)	421(23.2)	353 (19.6)	287 (15.9)	358 (20.0)
Total vegetable intake, servings/week ^4^	19.2 ± 0.3	20.1 ± 0.3	21.4 ± 0.3	21.4 ± 0.3	25.6 ± 0.3
Total fruit intake, servings/week ^4^	18.0 ± 0.4	18.8 ± 0.4	19.2 ± 0.3	19.2 ± 0.3	18.6 ± 0.4
History of disease (yes), *n* (%)					
Hypertension	393 (21.1)	293 (15.7)	268 (14.4)	221 (11.9)	198 (10.6)
Dyslipidemia	47 (2.5)	46 (2.5)	42 (2.3)	47 (2.5)	42 (2.3)
Diabetes	173 (9.3)	131 (7.0)	111 (6.0)	112 (6.0)	93 (5.0)

^1^ Values are mean ± standard error or *n* (%); ^2^ *p* values are derived from the χ^2^ test for categorical variables, and *p* for trends are derived from generalized linear regression analysis for continuous variables; ^3^ Physical activity level was calculated as metabolic equivalents (METs)-h/week and categorized as follow: Low, <20; Mid, ≥20 to <40; High, ≥40; ^4^ Total fruit and vegetable intake levels were adjusted for total energy intake with the standard multivariate method.

**Table 2:** Hazard ratios (95% confidence intervals) for cardiovascular disease (CVD) risk according to quintiles of consumption of total unprocessed meat and unprocessed red meat in Korean adults.

	Frequency of Consumption (Quintile)					*p* for Trend
	Q1	Q2	Q3	Q4	Q5	
	(*n* = 1862)	(*n* = 1865)	(*n* = 1863)	(*n* = 1859)	(*n* = 1862)	
Total unprocessed meat						
Median consumption, servings/week	0.46	1.15	1.93	3.08	5.55	
Case, *n*	147	106	78	82	73	
Model 1	1	0.77 (0.60–0.99)	0.61 (0.46–0.80)	0.72 (0.54–0.95)	0.71 (0.53–0.95)	0.05
Model 2	1	0.73 (0.56–0.95)	0.58 (0.43–0.78)	0.70 (0.51–0.96)	0.70 (0.50–0.99)	0.16
Model 3	1	0.72 (0.55–0.95)	0.57 (0.42–0.78)	0.69 (0.51–0.95)	0.69 (0.48–0.97)	0.14
Unprocessed red meat						
Median consumption, servings/week	0.29	0.86	1.49	2.36	4.50	
Case, *n*	147	99	87	70	83	
Model 1	1	0.73 (0.57–0.95)	0.70 (0.54–0.92)	0.65 (0.48–0.87)	0.76 (0.58–1.01)	0.12
Model 2	1	0.72 (0.55–0.94)	0.71 (0.53–0.95)	0.60 (0.43–0.83)	0.80 (0.58–1.12)	0.40
Model 3	1	0.72 (0.55–0.94)	0.71 (0.53–0.94)	0.60 (0.43–0.83)	0.79 (0.57–1.11)	0.38

Model 1: age-adjusted. Model 2: additionally adjusted for sex, total energy intake (kcal/day), body mass index (BMI) (BMI <18.5, 18.5–<23, 23–<25, ≥25), alcohol use (*n*/week), smoking (pack-years), physical activity (METs <20, 20–<40, ≥40), education status (≤elementary, middle school, high school, ≥university), household income (<1,000,000, 1,000,000–<2,000,000, 2,000,000–<4,000,000, ≥4,000,000 KRW), and residential area (urban, rural). Model 3: model 2 additionally adjusted for fruit and vegetable intake (servings/week).

**Table 3:** Hazard ratios (95% confidence intervals) for CVD risk according to quintiles of consumption for each type of unprocessed meat in Korean adults.

	Frequency of Consumption (Quintile)					*p* for Trend
	Q1	Q2	Q3	Q4	Q5	
	(*n* = 1862)	(*n* = 1865)	(*n* = 1863)	(*n* = 1859)	(*n* = 1862)	
Beef						
Median consumption, servings/week	0	0.06	0.12	0.23	0.75	
Case, *n*	178	46	115	69	72	
Model 1	1	1.15 (0.83–1.59)	0.95 (0.75–1.21)	0.91 (0.69–1.21)	0.81 (0.62–1.07)	0.1
Model 2	1	1.22 (0.86–1.71)	0.99 (0.77–1.27)	0.97 (0.72–1.30)	0.87 (0.64–1.19)	0.27
Model 3	1	1.21 (0.86–1.71)	0.99 (0.77–1.27)	0.96 (0.71–1.29)	0.86 (0.63–1.18)	0.26
Pork						
Median consumption, servings/week	0.23	0.63	1.14	1.9	3.65	
Case, *n*	143	106	89	66	82	
Model 1	1	0.93 (0.73–1.20)	0.81 (0.62–1.06)	0.64 (0.47–0.86)	0.86 (0.65–1.13)	0.14
Model 2	1	0.86 (0.66–1.13)	0.77 (0.57–1.03)	0.61 (0.44–0.84)	0.88 (0.63–1.22)	0.4
Model 3	1	0.86 (0.66–1.13)	0.76 (0.57–1.02)	0.60 (0.43–0.83)	0.87 (0.63–1.21)	0.38
Chicken						
Median consumption, servings/week	0	0.17	0.35	0.57	1.41	
Case, *n*	105	130	104	96	50	
Model 1	1	0.99 (0.77–1.29)	0.89 (0.68–1.17)	0.92 (0.69–1.22)	0.66 (0.47–0.93)	0.01
Model 2	1	0.98 (0.75–1.29)	0.90 (0.67–1.19)	0.99 (0.74–1.34)	0.69 (0.47–0.99)	0.049
Model 3	1	0.98 (0.75–1.29)	0.89 (0.67–1.19)	0.99 (0.74–1.34)	0.68 (0.47–0.99)	0.04
Organ meat						
Median consumption, servings/week	0	0.06	0.12	0.35		
Case, *n*	263	19	101	93		
Model 1	1	0.63 (0.39–1.02)	0.95 (0.76–1.20)	1.07 (0.84–1.36)		0.51
Model 2	1	0.64 (0.39–1.03)	0.98 (0.77–1.24)	1.04 (0.80–1.36)		0.7
Model 3	1	0.64 (0.39–1.04)	0.98 (0.77–1.25)	1.04 (0.80–1.36)		0.68

Model 1: age-adjusted. Model 2: additionally adjusted for sex, total energy intake (kcal/day), BMI (BMI <18.5, 18.5–<23, 23–<25, ≥25), alcohol use (*n*/week), smoking (pack-years), physical activity (METs <20, 20–<40, ≥40), education status (≤elementary, middle school, high school, ≥university), household income (<1,000,000, 1,000,000–<2,000,000, 2,000,000–<4,000,000, ≥4,000,000 KRW), and residential area (urban, rural). Model 3: model 2 additionally adjusted for fruit and vegetable intake (servings/week).
